# Structural basis for the recognition of HCoV-HKU1 by human TMPRSS2

**DOI:** 10.1038/s41422-024-00958-9

**Published:** 2024-04-19

**Authors:** Lingyun Xia, Yuanyuan Zhang, Qiang Zhou

**Affiliations:** 1 Center for Infectious Disease Research, Research Center for Industries of the Future, Zhejiang Key Laboratory of Structural Biology, School of Life Sciences, Westlake University. Institute of Biology, Westlake Institute for Advanced Study, Hangzhou, Zhejiang China; 2 Westlake Laboratory of Life Sciences and Biomedicine, Hangzhou, Zhejiang China

**Keywords:** Cryoelectron microscopy, Immunology

Dear Editor,

Human coronaviruses are pathogens capable of causing respiratory illnesses in humans, with seven identified species,^1^ three of which have caused epidemics or global pandemics in the past two decades, namely, Severe Acute Respiratory Syndrome Coronavirus (SARS-CoV),^2^ Middle East Respiratory Syndrome Coronavirus (MERS-CoV),^3^ and Severe Acute Respiratory Syndrome Coronavirus 2 (SARS-CoV-2).^4^ Along with these three viruses, HCoV-HKU1 (HKU1) belongs to the β-coronavirus genus. With three serotypes (HKU1-A, HKU1-B, and HKU1-C), HKU1 infection commonly causes the common cold, but also leads to severe lower respiratory tract infections.^5^ The spike (S) protein of HKU1 plays a crucial role in cell invasion by binding to host receptors, with the transmembrane serine protease TMPRSS2 recently identified as its protein receptor.^6^ TMPRSS2 comprises intracellular, transmembrane, LDL receptor A (LDLR-A, residues 112–149), scavenger receptor cysteine-rich (SRCR, residues 150–242), and C-terminal serine protease (SP, residues 256–489) domains. However, the structural basis of TMPRSS2 interaction with the S protein remains elusive. Here we present the cryo-electron microscopy (cryo-EM) structures of the HKU1-B S protein in the apo or receptor-bound states. The S protein of HKU1-B exhibits multiple conformations in the apo state, including a closed conformation and two active conformations. In the active conformations, one or two receptor-binding domains (RBD) of the S protein are in “up” position. Binding of the receptor TMPRSS2 results in more open conformations of the S protein, changing the interaction network in the S protein and triggering a trend towards the post-fusion state, thus facilitating the initiation of the invasion into host cells. Our research enhances the understanding of the HKU1 infection process, providing crucial insights for the development and optimization of vaccines and therapeutic interventions.

The S protein of HKU1-B forms a trimeric structure, with each protomer featuring a furin cleavage site “RRKRR”, akin to SARS-CoV-2. The S protein is cleaved into two subunits, S1 and S2. S1 comprises four domains: N-terminal lectin-like domain (NTD), RBD, subdomain 1 (SD1), and subdomain 2 (SD2). S2 includes the fusion peptide (FP), the heptad repeat regions 1 (HR1), the central helix (CH), the connector domain (CD), the heptad repeat regions 2 (HR2), the transmembrane domain (TM), and the C-tail domain (CT) (Fig. 1a). We first solved the structure of the HKU1-B S protein in the apo state using cryo-EM, revealing three conformations that included a closed conformation and two active conformations that contained one or two RBD domains in “up” position and named as “1up” or “2up”, respectively (Fig. 1b–d). For ease of description, unless specified otherwise in the following context, the term “S protein” refers to the spike protein of HKU1-B and the protomer containing the RBD in the “up” position of 1up conformation is defined as protomer 1, while the other two counterclockwise protomers are referred to as protomers 2 and 3, respectively (Fig. 1e). The overall resolutions for these conformations are 2.80 Å, 2.99 Å, and 3.16 Å, respectively (Supplementary information, Figs. S1 and S2, and Table S1). Remarkably, the apo state of the HKU1-B S protein differs from that of HKU1-A or HKU1-C, as the S proteins of HKU1-A^7^ or HKU1-C^8^ have only been isolated in the closed conformation. The closed conformations of the HKU1-B, the HKU1-A, and the HKU1-C S proteins are overall similar, except that the closed conformation of the HKU1-B S protein displays a slight counterclockwise rotation and a subtle outward tilt of NTD, representing a more compact closed conformation than the HKU1-C S protein (PDB ID: 5I08) (Supplementary information, Fig. S3). The RBD of HKU1-B (residues 320–610) is one of the largest among β coronaviruses (Supplementary information, Fig. S4). In contrast to SARS-CoV-2, the RBD of HKU1-B contains longer RL1 loop (residues 430–457) and RL2 loop (residues 466–537), while RL3 loop (residues 548–574) has a comparable length (Supplementary information, Fig. S4). The RL2 occupies a spatial position corresponding to the receptor-binding motif (RBM) of SARS-CoV-2 RBD, indicating its potential for receptor binding.^9^ The sequence identity between the RBDs of HKU1-A and HKU1-B is 74%, while that between HKU1-B and HKU1-C reaches 98%, with only sporadic mutations observed at the RL3 site (Supplementary information, Fig. S5). In the closed conformation of the S protein, these loops engage in interactions with adjacent RBDs and NTDs, stabilizing the structure of the S protein (Supplementary information, Fig. S6).

**Fig. 1:**
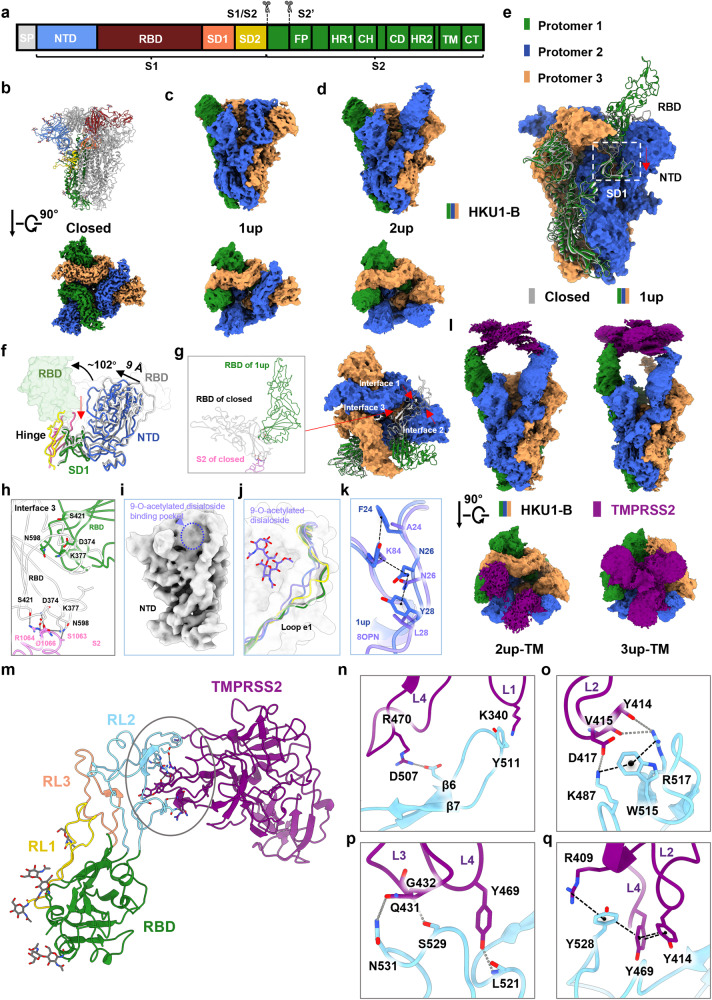
Cryo-EM structures of the HKU1-B S protein alone or in complex with TMPRSS2. **a** A diagram representation of the domains of the HKU1-B S protein. S1/S2 and S2′ are the protease cleavage sites. SP signal peptide, NTD N-terminal domain, RBD receptor-binding domain, SD1 subdomain 1, SD2 subdomain 2, FP fusion peptide, HR1 heptad repeat 1, CH central helix, CD connector domain, HR2 heptad repeat 2, TM transmembrane region, CT cytoplasmic tail. **b** The structure shown in side view (upper) and the cryo-EM map show in top view (lower) of the HKU1-B S protein in closed conformation. One of protomers in structure is colored by domains as in **a**. **c**, **d** Cryo-EM maps of the HKU1-B S protein in 1up (**c**) or 2up (**d**) conformations shown in side view (upper) or top view (lower). **e** Comparison of the 1up conformation and the closed conformation (gray) of the HKU1-B S protein, with the protomers of 1up conformation colored in forest (protomer 1), marine (protomer 2), and orange (protomer 3), respectively. White dashed box: the movement direction of SD1 from the closed conformation to the 1up conformation of the S protein is indicated by red arrow. **f** Conformational changes of the HKU1-B S protein between closed conformation and 1up conformation. The hinge region between the RBD and SD1 of HKU1-B S protein in 1up or closed conformation is colored in magenta or yellow, and the remaining colors are consistent with **e**. The black curved arrows indicate the rotation direction of the RBD; black straight arrows indicate the movement direction of the NTD; and red straight arrows indicate the movement direction of SD1 (all from closed conformation to 1up conformation). **g** Same as **e** but shown in top view, with the interface 1 between RBD of protomer 1 and RBD of protomer 2 and the interface 2 between RBD of protomer 1 and NTD of protomer 2 indicated by red arrows in the closed conformation. The interface 3 between RBD and S2 subunit of protomer 1 is shown in inset. **h** Opening of RBD disrupts its interface with the S2 subunit of the same protomer. **i** No apparent cryo-EM density observed in the 9-O-acetylated disialoside-binding pocket in NTD of the 1up conformation. **j** Comparison of the different conformations of the e1 loop. The e1 loop of the NTD adjacent to the RBD in “up” position (PDB ID: 8OPN) or “down” position (PDB ID: 8OHN) of the HKU1-A S protein is shown in light purple or yellow, respectively. The e1 loop of the HKU1-B S protein in closed conformation is shown in gray. The e1 loop of the NTD (protomer 2) adjacent to the RBD in “up” position and the e1 loop of the NTD (protomer 3) adjacent to the RBD in the “down” position of the HKU1-B S protein in 1up conformation are shown in marine and forest, respectively. **k** Overlay of the e1 loop of the NTD (protomer 2) adjacent to the RBD in “up” position of the HKU1-B S protein in 1up conformation (marine) with that of the HKU1-A S protein in 1up conformation (light purple) with the key residues maintaining the conformation shown in stick. **l** Cryo-EM maps of the HKU1-B S protein in complex with TMPRSS2. **m**–**q** Interactions between HKU1-B RBD and TMPRSS2. The trypsin-like serine peptidase domain of TMPRSS2 is predominantly recognized by HKU1-B RBD (**m**). Detailed interactions on the interface between HKU1-B RBD and TMPRSS2 (**n**–**q**).

Compared to the closed conformation, the RBD of protomer 1 undergoes a rotation of 102° around the SD1 domain in the 1up conformation (Fig. 1f). The interfaces between the “up” position RBD and the other regions are reshaped, losing interactions with the RBD, NTD, and HR1 of protomer 2 (Fig. 1g, h; Supplementary information, Videos S1 and S2). The 1up conformation of the S protein trimer exhibits a clockwise rotation of 2° (Supplementary information, Fig. S7), with the NTD of protomer 2 moving inward by 9 Å relative to the closed conformation (Fig. 1f). The SD1 of protomer 1 moves along with the NTD of protomer 2 (Fig. 1f; Supplementary information, Video S2), maintaining the contact interface mediated by a hydrogen bond (H-bond) network and π interactions (residues 50–56 of protomer 2 and residues 644–650 of protomer 1). These conformational changes reshape the contact interface of the RBDs, facilitating their release and activation.

Recent research suggests that the NTD of HKU1-A binds to the host cell 9-O-acetylated disialoside, triggering the opening of S1.^7^ In the two active conformations of the HKU1-B S protein, there is no apparent additional cryo-EM density in the pocket (Fig. 1i). In the 1up conformation, the e1 loop (residues 29–37) of NTD contacted by the “down” RBD of HKU1-B resembles the disialoside-free conformation of the HKU1-A S protein (Fig. 1j). Meanwhile, the e1 loop of NTD contacted by the “up” RBD undergoes an inward shift similar to the disialoside-bound conformation of the HKU1-A S protein (Fig. 1j). The Y28 (corresponding to L28 in HKU1-A), N26, and F24 (corresponding to A24 in HKU1-A) in the e1 loop form interactions together with Y84 (corresponding to K84 in HKU1-A), contributing to stabilizing the conformation of the e1 loop (Fig. 1k).

To unravel the mechanism underlying the interaction between the S protein of HKU1-B and the host cell receptor TMPRSS2, we elucidated the cryo-EM structures of the HKU1-B S protein incubated with TMPRSS2 in various conformations, including a closed conformation, two 1up conformations (designated as 1up-1 and 1up-2), two 2up conformations (with or without TMPRSS2 bound, referred to as 2up-TMR and 2up-1), and a 3up conformation with TMPRSS2 bound (referred to as 3up-TMR) (Fig. 1l; Supplementary information, Figs. S8, S9 and S10, and Table S2). Both 1up-1 and 1up-2 conformations contain an RBD in “up” position but with different rotation angles. The 1up-1 conformation is reminiscent of the previously mentioned 1up conformation of the S protein (Supplementary information, Fig. S11a). In the 1up-2 conformation, the “up” position RBD of protomer 1 exhibits a larger angle and loses its interface with the adjacent RBDs of neighboring protomers 2 and 3 (Supplementary information, Fig. S11b) which is likely attributable to the influence exerted by TMPRSS2. The 2up-1 conformation is similar to the previously mentioned 2up conformation of the S protein (Supplementary information, Fig. S11c). In the 2up-TMR conformation, two RBDs adopt the “up” position, each bound with a TMPRSS2 molecule, while the remaining RBD of protomer 3 remains in the “down” position. In 2up-TMR conformation, the RBD of protomer 3 disengages from the interface with the “up” position RBD of protomer 1, potentially contributing to the subsequent generation of the 3up conformation. (Supplementary information, Fig. S11d and Video S3). In the 3up-TMR conformation, all three RBDs are fully in “up” position, each bound with a TMPRSS2 molecule. Compared to the 2up-TMR conformation, the 3up-TMR rotates clockwise by 8°, with its RBDs leaning more towards the central axis. In a counterclockwise sequence (protomer 1-2-3), the magnitude of conformational changes of RBDs and NTDs gradually decreases, with minimal change observed in the NTD of protomer 3 (Supplementary information, Fig. S11e and Video S3). The three TMPRSS2 molecules form interfaces maintained by cation–π and π–π interactions (Supplementary information, Fig. S11f). Additionally, in the 3up-TMR conformation, the CH bundle shifts outward by 2 Å compared to the 2up-TMR conformation (Supplementary information, Fig. S11g), indicating the efficacy of the receptor binding on the modulation of the S protein conformation and subsequent membrane fusion.^10^

To improve the local resolution of the interface region, we employed local refinement for the 3up-TMR conformation, achieving a local resolution of 3.6 Å, allowing reliable interface modeling and analysis (Supplementary information, Fig. S12). The RBD primarily binds to the TMPRSS2 molecule through polar interactions. The RL2 loop of the RBD firmly clasps the SP domain of TMPRSS2 like a clamp, near the active pocket of SP domain, creating an interface of 850 Å^2^ (Fig. 1m). On one side of the clamp, the RL2 loop of the RBD interacts with the loop1, loop2 and loop4 of TMPRSS2 through intensive interactions (Fig. 1n, o). The Lys487 and Asp507 of the RL2 loop form salt bridges with Asp417 and Arg470 of TMPRSS2, respectively. The Try511 of the RL2 loop interacts with Lys340 of TMPRSS2 through cation–π interaction. There are H-bonds formed between Arg517 of the RL2 loop and Tyr414 and Val415 of TMPRSS2. Lys487 and Arg517 of the RBD are stabilized by Trp515 of the RBD through cation–π interaction (Fig. 1o), which is crucial for receptor recognition and binding.^6^ On the other side of the clamp, Leu521, Ser529, and Asn531 in the RL2 loop interact with Tyr469, Gly432, and Gln431 of TMPRSS2 through H-bonds (Fig.1p). Tyr528 of the RL2 loop and Arg409, Tyr469, and Tyr414 of TMPRSS2 form an interaction string involved cation–π interaction and π–π stacking (Fig. 1q). The residues of TMPRSS2 involved in binding to HKU1-B lack conservation across the TMPRSS family (Supplementary information, Fig. S13), providing an explanation for previous findings that other members of the TMPRSS family cannot serve as receptors for the HKU1.^6^

The HKU1-B RBD exhibits an overall similar structure in the closed, active, and TMPRSS2-bound conformation, with certain regions undergoing substantial displacement and rotation, particularly the β hairpin (β9/β10) region. Tyr511 is notably rotated 5 Å upward, forming a bond with Lys340, revealing the impact of receptor binding on the structural changes of the HKU1-B RBD (Supplementary information, Fig. S14).

There are some variations in the interaction interface of the S proteins of HKU1-A or HKU1-C with TMPRSS2 (Supplementary information, Fig. S5). In HKU1-A RBD, the mutations, such as K487S, Y511D, and D507T, disrupt polar interactions with TMPRSS2 at the upper interface of the clamp. Additionally, the mutations N531R and S529D on the other side of the clamp may affect the interaction of HKU1-A RBD with TMPRSS2. And all of these mutations may explain the changes in the affinity of TMPRSS2 for the RBDs of both HKU1-A and HKU1-B.^6^

The transition of the S protein to the open conformations, which facilitates the binding of its RBD to the receptor, is crucial for the viral invasion mediated by the S protein.^10^ The S proteins of HKU1-A and HKU1-C are found in closed conformations in the apo state.^7,8^ In this study, we discover that the S protein of HKU1-B exhibits both closed and open conformations in the absence of the receptor, akin to SARS-CoV, SARS-CoV-2, and MERS-CoV.^11,12^ The ability of the HKU1-B S protein to spontaneously adopt active conformations highlights the difference between the HKU1-B and HKU1-A or HKU1-C. Our research suggests that the binding of the receptor TMPRSS2 can induce the S protein to more open conformations. The binding of TMPRSS2 induces a clockwise rotation of the S1 subunit and a centripetal compression of the NTD, resulting in the rise of RBDs and reducing the contact area between S1 and S2. The receptor-driven conformational changes in the HR1 and CH region of the S2 subunits at this point may provide a conformational basis for triggering the dramatical conformation change of the S2 subunits that cause membrane fusion and the viral invasion process.^13^

Coronaviruses employ various strategies to bind host cells to enhance their invasion efficiency. The S protein of HKU1-A binds to non-protein receptor 9-O-acetylated disialoside, a sialylated polysaccharide,^14^ triggering its conformational changes and resulting in RBDs in “up” position that favors the binding of the protein receptor,^7^ reflecting the synergistic interactions between non-protein and protein receptors, and promoting the efficiency of viral invasion into host cells.

### Supplementary information

Supplementary Information

Supplementary information, Video S1

Supplementary information, Video S2

Supplementary information, Video S3

## Data Availability

Atomic coordinates and cryo-EM maps (PDB ID: 8Y19, 8Y1A, 8Y1B, 8Y1C, 8Y1D, 8Y1E, 8Y1F, 8Y1G, 8Y1H; EMDB ID: EMD-38828, EMD-38829, EMD-38830, EMD-38831, EMD-38832, EMD-38833, EMD-38834, EMD-38835, EMD-38836) have been deposited to the Protein Data Bank (http://www.rcsb.org) and the Electron Microscopy Data Bank (https://www.ebi.ac.uk/pdbe/emdb/), respectively.
