# Supplementary material for: Structural basis for the recognition of HCoV-HKU1 by human TMPRSS2
Source: Cell Res. 2024 Apr 19;34(7):526–9. doi: 10.1038/s41422-024-00958-9 (PMC11217304; doi:10.1038/s41422-024-00958-9)
Supplement: Supplementary file 1 — Supplementary Information [file 41422_2024_958_MOESM1_ESM.pdf]

Supplementary information

Structural basis for the recognition of HCoV-HKU1 by human TMPRSS2

Lingyun Xia[1,2†], Yuanyuan Zhang[1,2†], Qiang Zhou[1,2*]

[1]Center for Infectious Disease Research, Research Center for Industries of the Future, Zhejiang Key Laboratory of Structural Biology, School of Life Sciences, Westlake University; Institute of Biology, Westlake Institute for Advanced Study, Hangzhou, Zhejiang Province, China.

[2]Westlake Laboratory of Life Sciences and Biomedicine, Hangzhou, Zhejiang Province, China.

[†]These authors contributed equally to this work.

[*]Corresponding author. Email: zhouqiang@westlake.edu.cn

Materials and Methods

Protein preparation

The extracellular domain (ECD) (1-1290 a. a.) of S protein of HKU1-B (uniport ID: Q14EB0) was cloned into the pCAG vector (Invitrogen) with two proline substitutions at residues 1067 and 1068 and a C-terminal T4 fibritin trimerization motif followed by one Flag tag and a 10xHis tag. This construct will hereafter be referred to as S. A "GSAS" mutation at residues 752 to 755 was introduced into S to prevent the host furin protease digestion. The extracellular domain (109-492 a. a.) of human TMPRSS2 (accession number: NM_005656.4) was also cloned into the pCAG vector (Invitrogen) with an N-terminal signal peptide of secreted luciferase and a C-terminal 10xHis tag. This construct will hereafter be referred to as TMR. The "Q" mutation at residue S255 was introduced into TMR to prevent the autodigestion. The mutants were generated with a standard two-step PCR based strategy. All the plasmids used to transfect cells were prepared by GoldHi EndoFree Plasmid Maxi Kit (CWBIO). The purification processes of the S were same as the S protein of SARS-CoV-2 that we have been described previously. The S protein and TMPRSS2 were purified as below: The recombinant proteins were overexpressed using the HEK293F mammalian cells (Invitrogen) at 37°C under 5% CO_2 in a Multitron-Pro shaker (Infors, 130 rpm). When the cell density reached 2.0×10^6 cells/mL, the plasmid was transiently transfected into the cells. To transfect one liter of cell culture, about 1.5 mg of the plasmid was premixed with 3 mg of polyethylenimines (PEIs) (Polysciences) in 50 mL of fresh medium for 30 min before adding to cell culture. Cells were removed by centrifugation at 4000×g for 15 min after seventy hours transfection. The secreted proteins were purified by Ni-NTA Agarose (GE Healthcare). After loading two times, the Ni-NTA Agarose was washed with the wash buffer containing 25 mM Tris (pH 8.0), 150 mM NaCl, 50 mM imidazole. The protein was eluted with the elute buffer containing 25 mM Tris (pH 8.0), 150 mM NaCl, 500 mM imidazole. The eluent of TMR was then concentrated and subject to size-exclusion chromatography (Superdex 200 Increase 10/300 GL, GE Healthcare) in buffer containing 25 mM Tris (pH 8.0), 150 mM NaCl. The eluent of S protein was subject to size-exclusion chromatography (Superose 6 Increase 10/300 GL, GE Healthcare) in buffer containing 25 mM Tris (pH 8.0), 150 mM NaCl and the peak fractions were collected for EM analysis and generation of S -TMR complex. The S protein was incubated with TMPRSS2 at a molar ratio of about 1: 5 for one hour. Then the mixture was subject to size-exclusion chromatography (Superose 6 Increase 10/300 GL, GE Healthcare) in buffer containing 25 mM Tris (pH 8.0), 150 mM NaCl. The peak fractions were collected and concentrated for EM analysis.

Cryo-EM sample preparation and data acquisition

S protein alone and S-TMR complex were concentrated to ~2.5 mg/mL and applied to the grids, respectively. Aliquots (3.5 μL) of the protein were placed on glow-discharged holey carbon grids (Quantifoil Au R1.2/1.3). The grids were blotted for 3.5 s and flash-frozen in liquid ethane cooled by liquid nitrogen with Vitrobot (Mark IV, Thermo Scientific). The prepared grids were transferred to a Titan Krios operating at 300 kV equipped with a Gatan K3 detector and GIF Quantum energy filter. Movie stacks were

automatically collected using EPU software (Thermo Fisher Scientific), with a slit width of 20 eV on the energy filter and a defocus range from –1.2 µm to –2.2 µm in super-resolution mode at a nominal magnification of 81,000×. Each stack was exposed for 2.56 s with an exposure time of 0.08 s per frame, resulting in a total of 32 frames per stack. The total dose was ~50 $e^-/Å^2$ for each stack.

Data processing

The movie stacks were motion corrected with MotionCor2[1] and binned twofold, resulting in a pixel size of 1.087 Å/pixel. Meanwhile, dose weighting was performed[2]. After patch CTF estimation, particles were automatically picked according to the templates generated from an initial 2D classification of manually picked particles by cryoSPARC[3]. After 2D classification, the particles with clear secondary structure features were selected and subjected to ab-initio reconstruction to obtain the initial models, then multi-hetero refinement without symmetry were performed to selected good particles using cryoSPARC[3]. The selected particles were subjected to non-uniform refinement, local CTF refinement and local refinement with C1 symmetry, resulting in the 3D reconstruction for the whole structures, which was further subject to local refinement with an adapted mask on the interface between RBD of S protein and TMPRSS2 to improve the map quality on RBD-TMR subcomplex. The resolution was estimated with the gold-standard Fourier shell correlation 0.143 criterion[4] with high-resolution noise substitution[5]. Refer to Figs. S2, S3, S6 and S7 and Tables S1 and S2 for details of data collection and processing.

Model building and structure refinement

For the model building of S protein, the predicted atomic model by Alphafold 2[6,7] were used as templates, which were molecular dynamics flexible fitted[8] into the whole cryo-EM map and manually adjusted with Coot[9] to obtain the atomic model of S protein in different states. For the S-TMR complex, the model building was accomplished based on the focused refined. For the S-TMR the predicted atomic model of TMPRSS2 (Alphafold ID: AF-O15393-F1)[6] [7]was used as template. Each residue was manually checked with the chemical properties taken into consideration during model building. Several segments, whose corresponding densities were invisible, were not modeled. Structural refinement was performed in Phenix[10] with secondary structure and geometry restraints to prevent overfitting. Statistics associated with data collection, 3D reconstruction and model building were summarized in Tables S1 and S2.

References

1 Zheng, S. Q. *et al. Nat Methods* **14**, 331-332 (2017).

2 Grant, T. & Grigorieff, N. *Elife* **4**, e06980 (2015).

3 Punjani, A. *et al. Nat Methods* **14**, 290-296 (2017).

4 Rosenthal, P. B. & Henderson, R. *J Mol Biol* **333**, 721-745 (2003).

5 Chen, S. *et al. Ultramicroscopy* **135**, 24-35 (2013).

6 Jumper, J. *et al. Nature* **596**, 583-589 (2021).

7 Varadi, M. *et al. Nucleic Acids Research* **50**, D439-D444 (2021).

8 Trabuco, L. G. *et al.* *Structure* **16**, 673-683 (2008).
9 Emsley, P. *et al.* *Acta Crystallogr D Biol Crystallogr* **66**, 486-501 (2010).
10 Adams, P. D. *et al.* *Acta Crystallogr D Biol Crystallogr* **66**, 213-221 (2010).

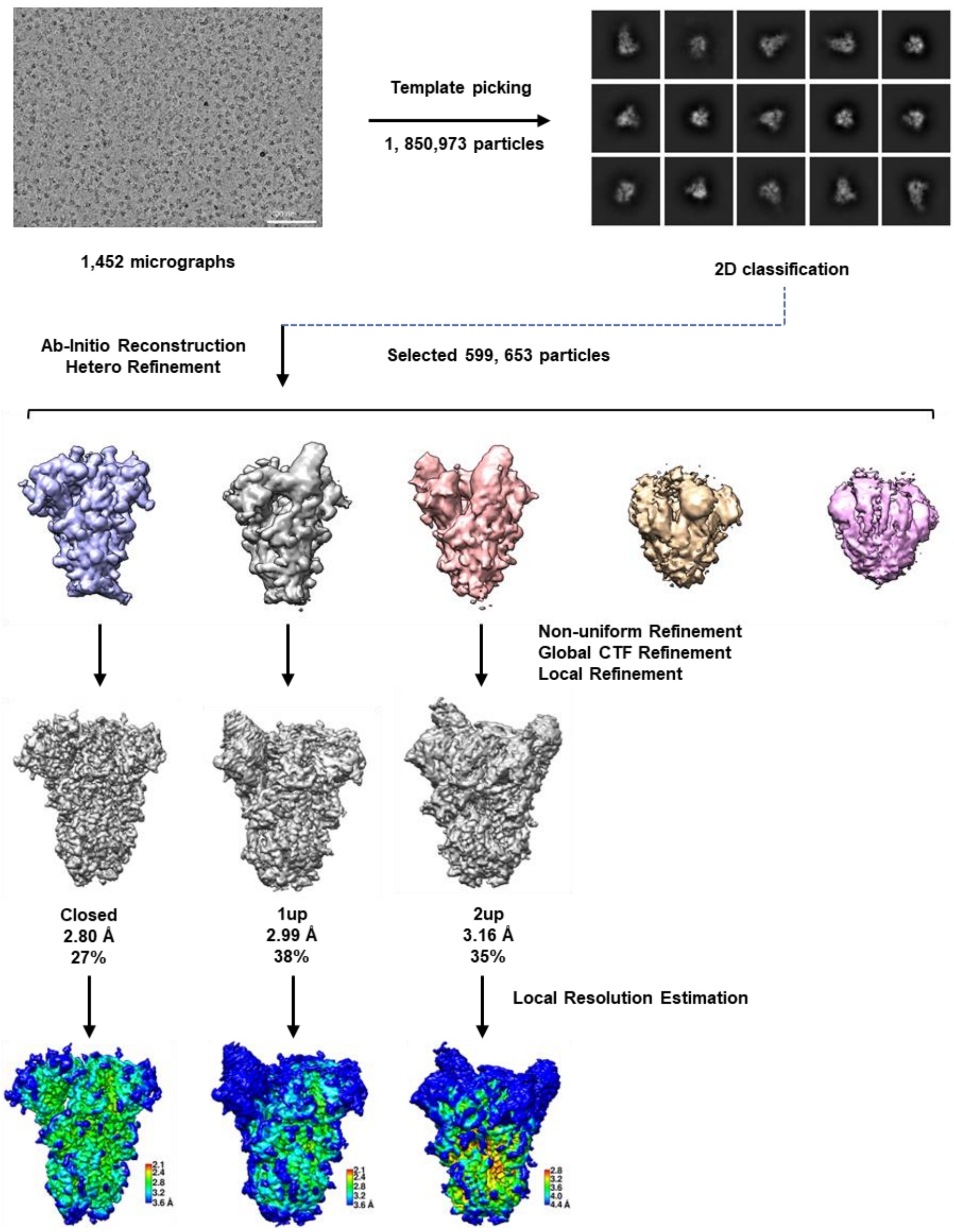

Fig. S1. Cryo-EM data processing pipeline for the apo HKU1-B S protein.

Please see the "Data Processing" section in Methods for details.

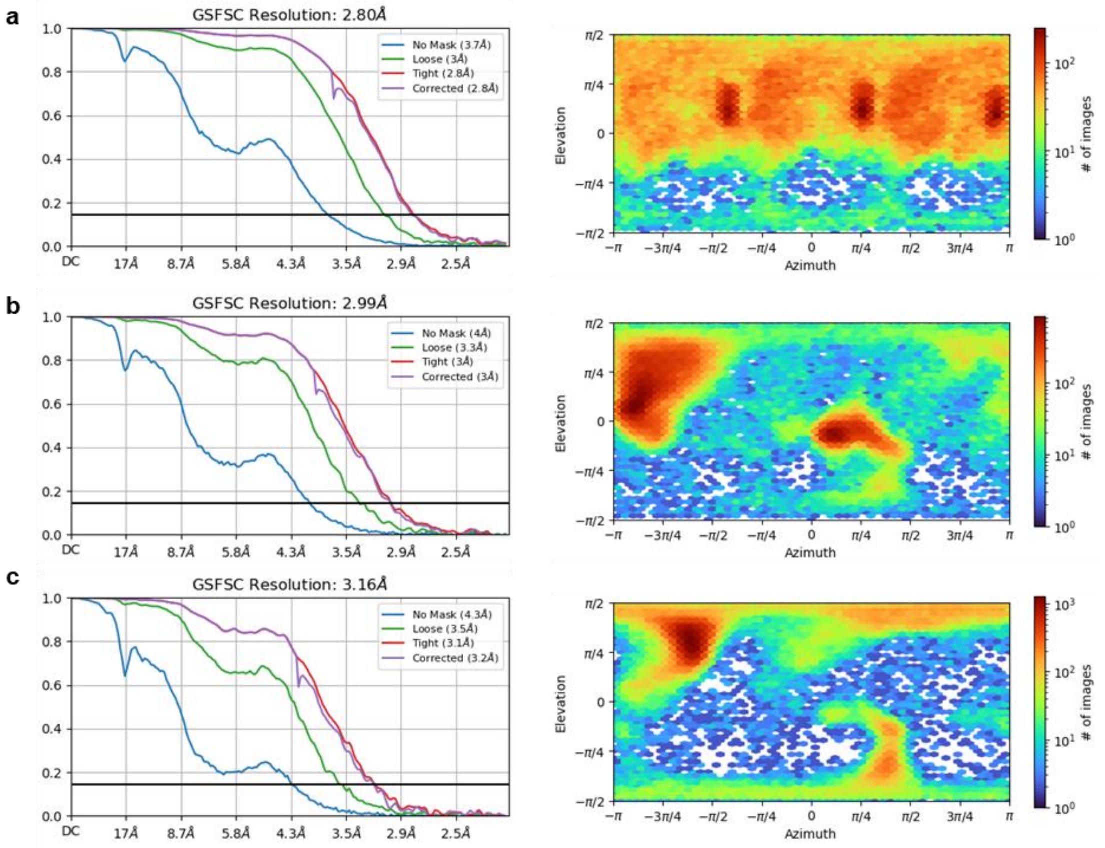

Fig. S2. Cryo-EM analysis of the apo HKU1-B S protein

a-c, Euler angle distribution of the apo HKU1-B S protein in the final 3D reconstruction in cryoSPARC (right). Gold standard FSC curve of the apo HKU1-B S protein is estimated by cryoSPARC (left).

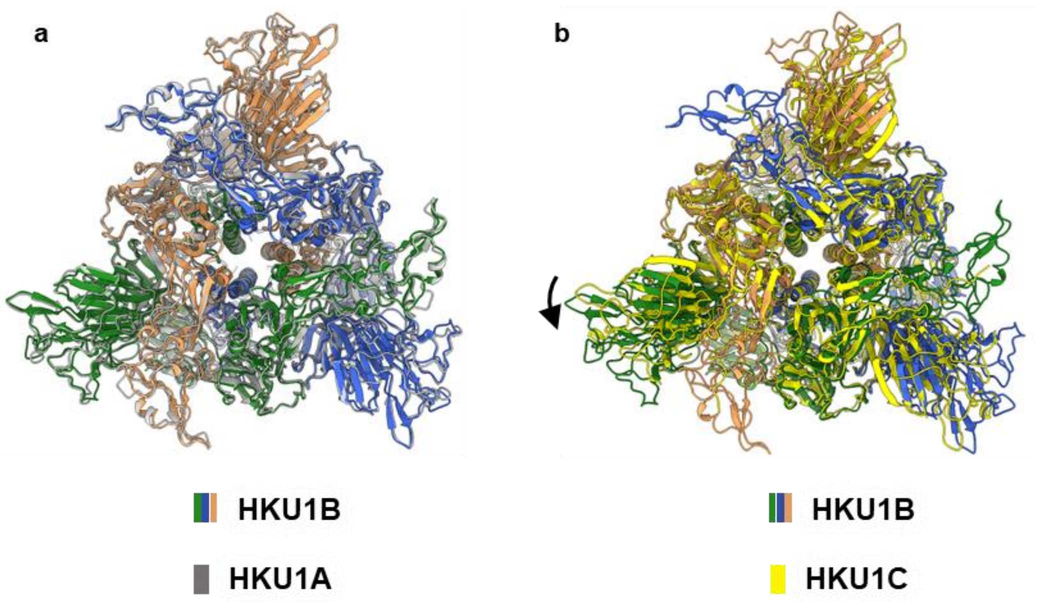

Fig. S3. Compares the structures of the S proteins of HKU1-A/B/C.

Structural comparison of the S proteins of HKU1-B with HKU1-A (a) or HKU1-C (b). The structures of HKU1-A and HKU1-B are represented by PDB IDs 8OHN and 5I08, respectively. The arrow indicates the HKU1-B S protein displays a slight counterclockwise rotation compared to the HKU1-C S protein.

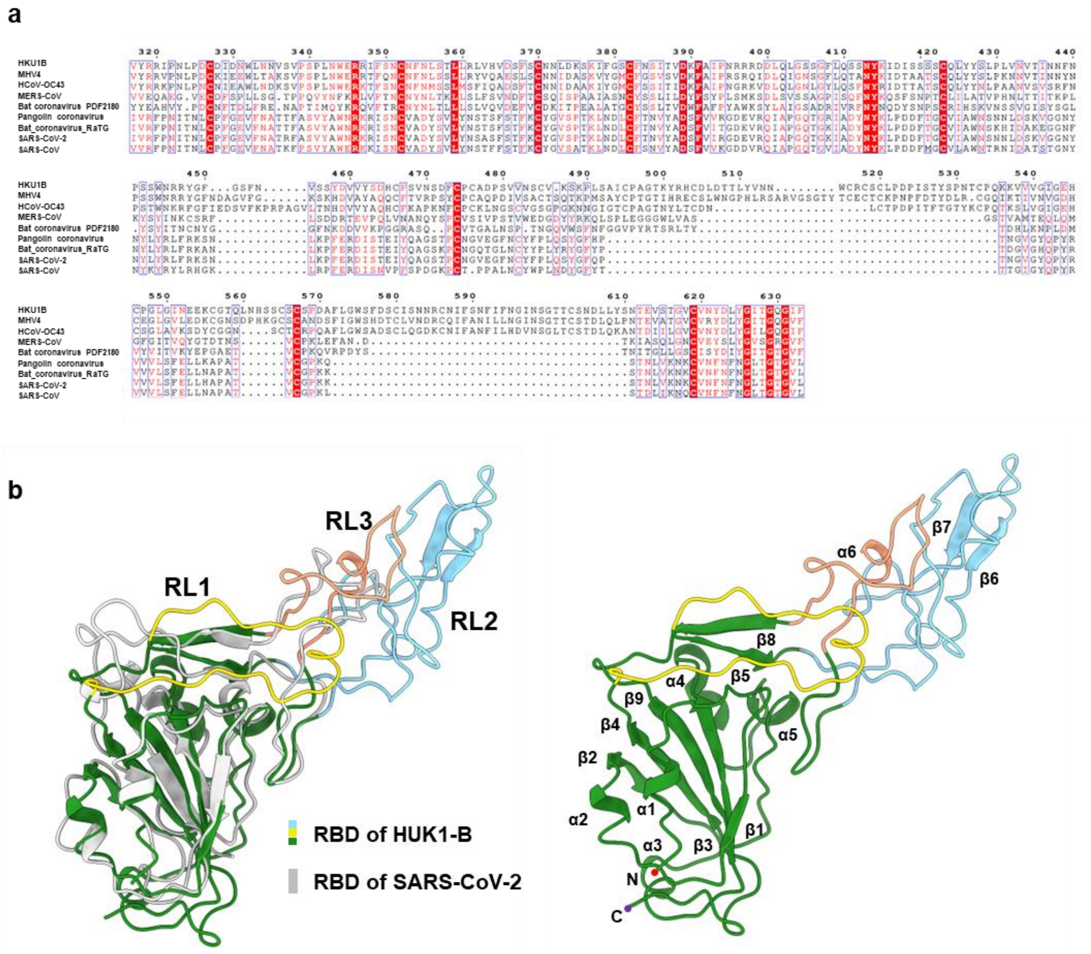

Fig. S4. Comparison of RBD structures in beta coronaviruses.

a, Sequence alignment of beta coronavirus RBDs. **b,** Structural comparison of RBDs between HKU1-B and SARS-CoV-2 (PDB ID: 6M17). The genomic sequence of Bat coronavirus PDF-2180 is sourced from GenBank Accession numbers NC_034440.1. Uniport numbers for other beta coronavirus S proteins are: P0DTC2 (SARS-CoV-2), P22432 (MHV-4), P36334 (HCoV-OC43), P59594 (SARS-CoV), A0A6B9WHD3 (Bat coronavirus RaTG13), K9N5Q8 (MERS-CoV), and A0A6G6A1M4 (Pangolin coronavirus).

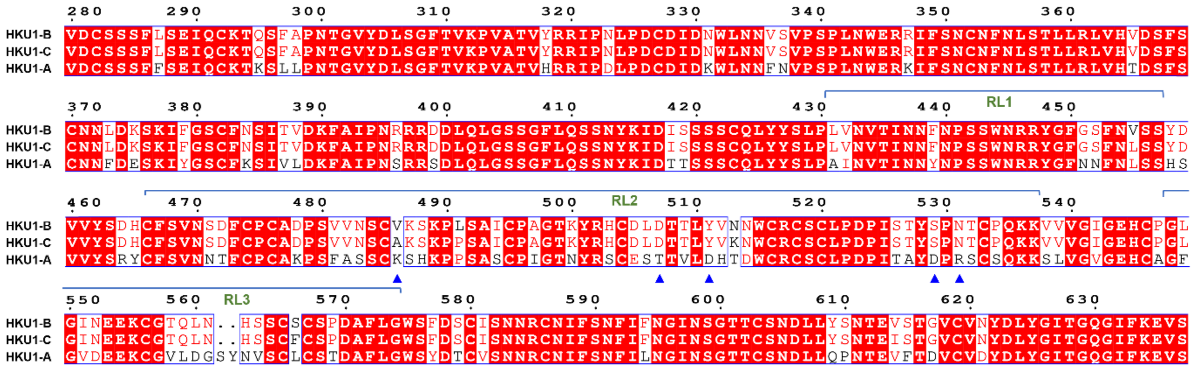

Fig. S5. Sequence alignment of HKU1-B, HKU1-A and HKU1-C RBD.

The blue triangle subscripts indicate polar residues at the interface of HKU1-B RBD binding to TMPRSS2 that have undergone mutations in HKU1-A RBD.

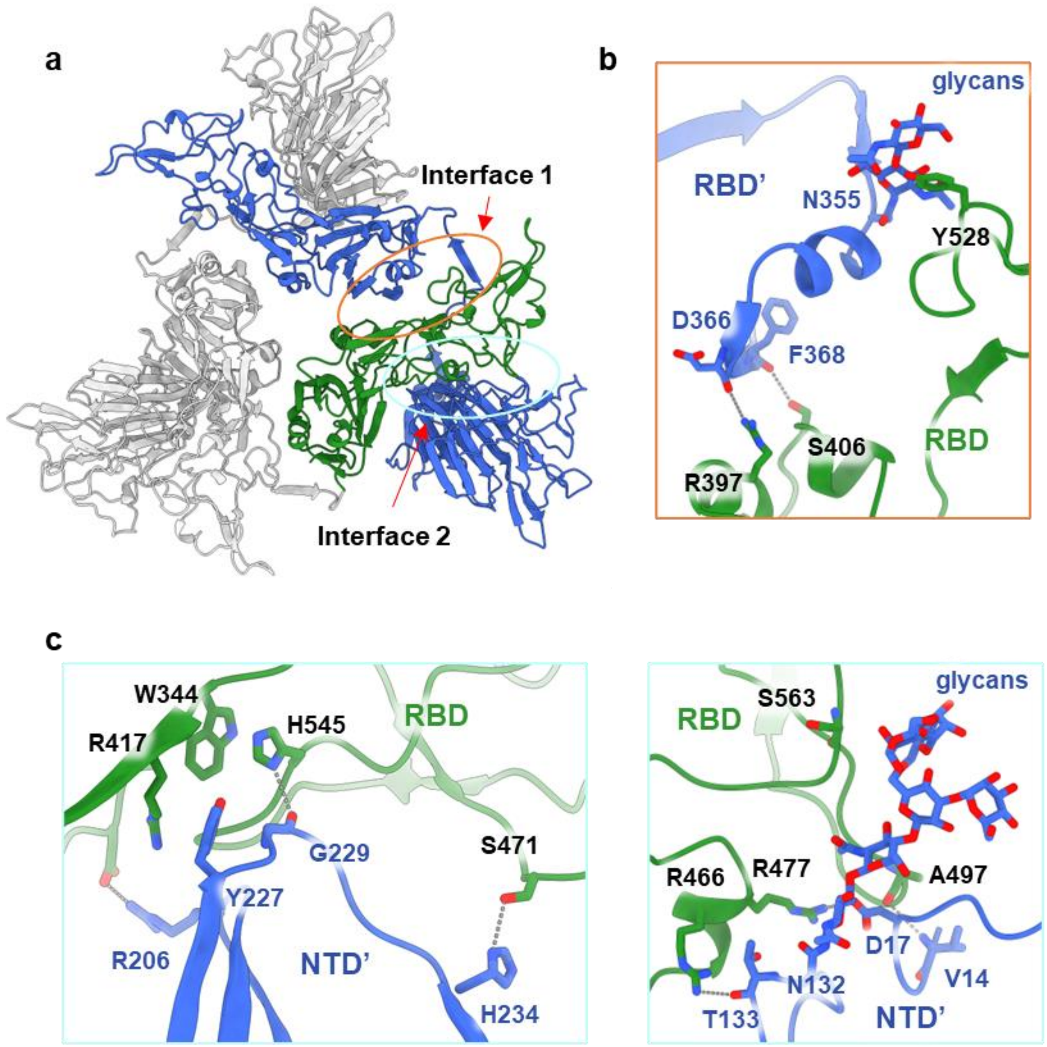

Fig. S6. Interactions maintaining the closed conformation of the HKU1-B S protein.

a, Top view of the closed conformation of HKU1-B S protein, colored counterclockwise for each protomer of the closed conformation in forest (protomer 1), marine (protomer 2), and yelloworange (protomer 3), respectively. **b** to **c** Contact interfaces of the closed conformation RBD with adjacent protomers, including interfaces with neighboring RBD (red circle) and adjacent NTD (blue circle).

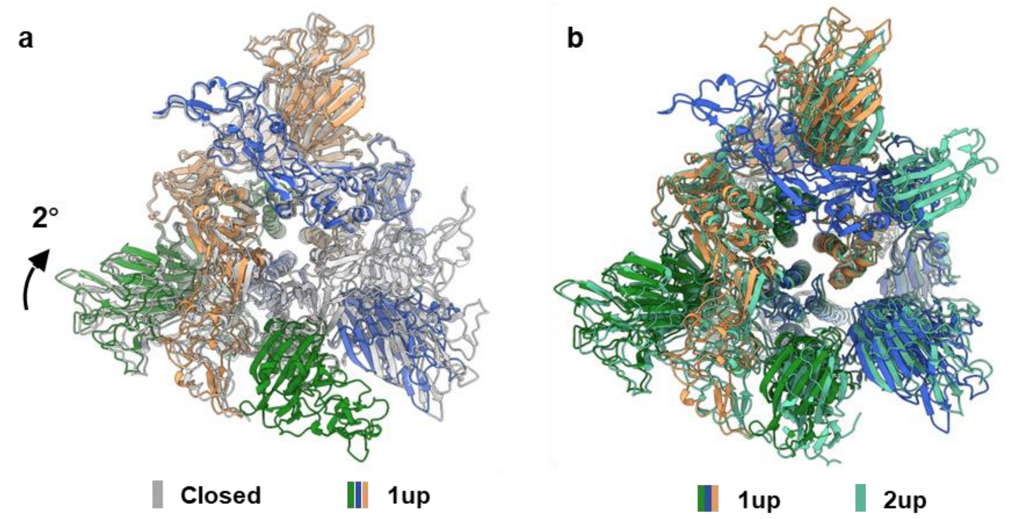

Fig. S7. Conformational changes in HKU1-B S protein.

a, Structural comparison between 1up and closed conformations of the S protein in HKU1-B. **b,** Structural comparison between 2up and 1up conformations of the S protein.

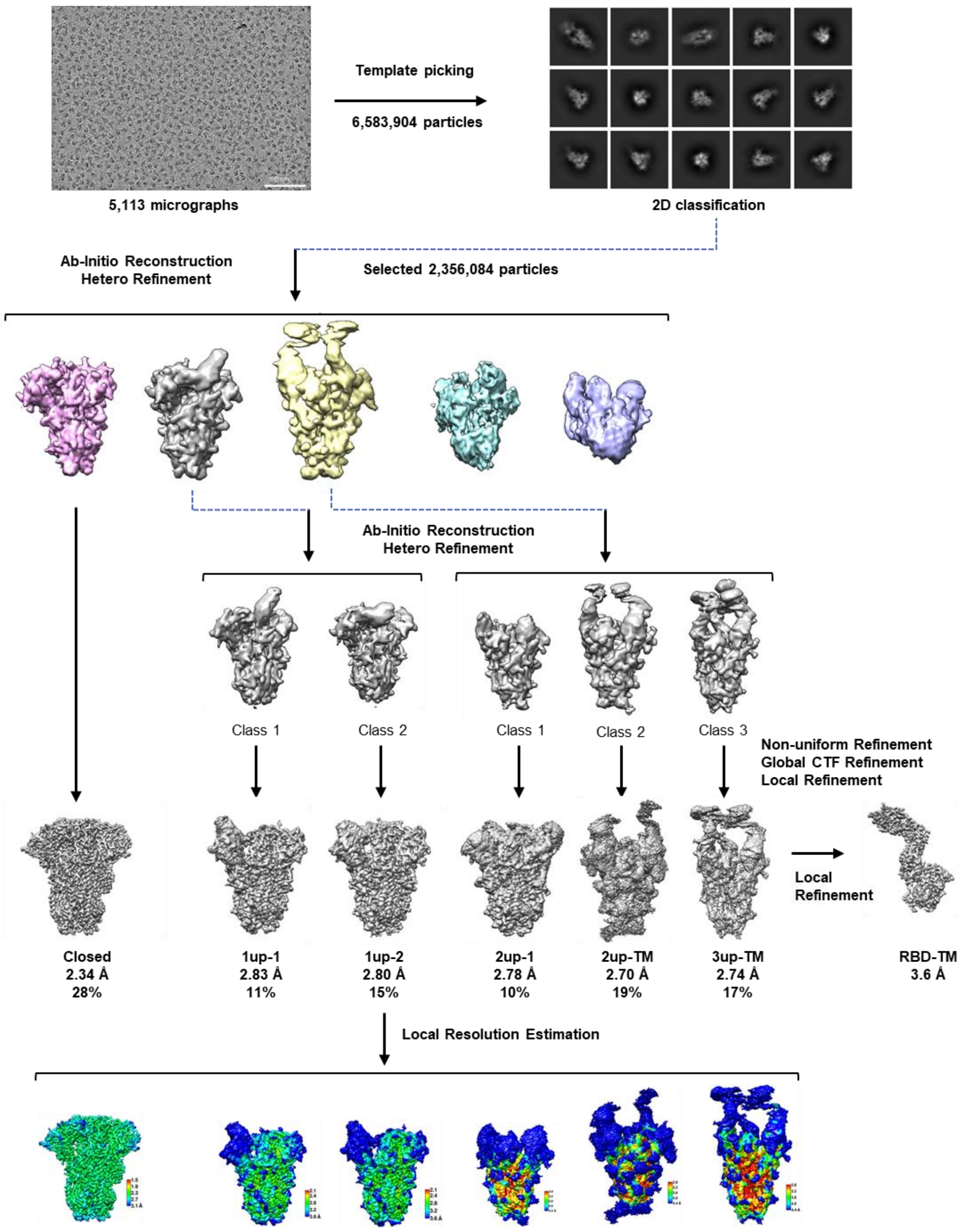

Fig. S8. Cryo-EM data processing pipeline for the complex of HKU1-B S protein and TMPRSS2.

Please see the "Data Processing" section in Methods for details.

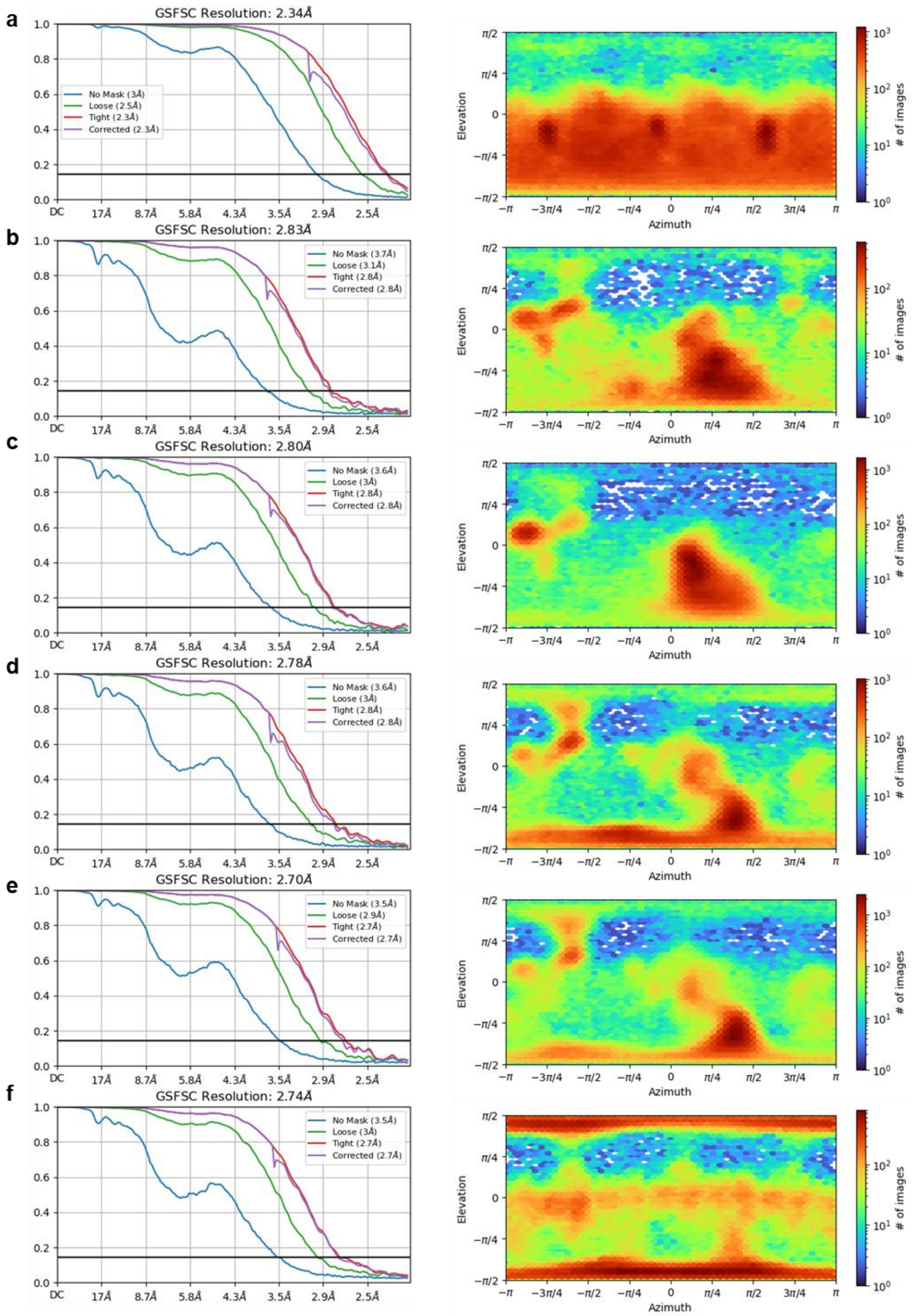

Fig. S9. Cryo-EM analysis of the apo HKU1-B S protein

a-f, Euler angle distribution of the apo HKU1-B S protein in the final 3D reconstruction in cryoSPARC (right). Gold standard FSC curve of the apo HKU1-B S protein is estimated by cryoSPARC (left).

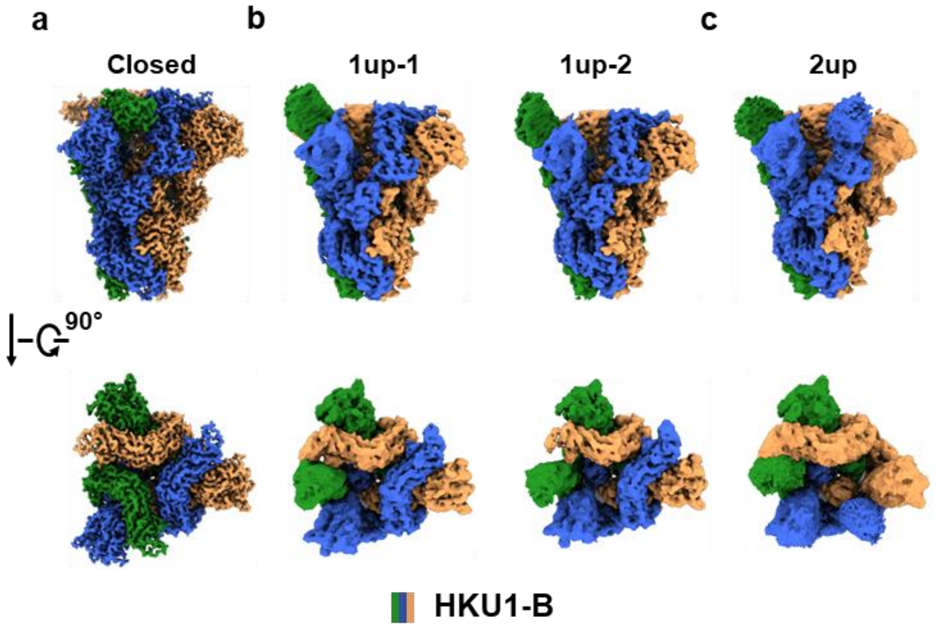

Fig. S10. Cryo-EM maps of the HKU1-B S protein incubated with TMPRSS2. Orthogonal views are shown for the closed conformation (**a**), the two 1up conformations (**b**), and the two 2up conformations (**c**). The three protomers of the HKU1-B S protein are colored in forest (protomer 1), marine (protomer 2), and yelloworange (protomer 3).

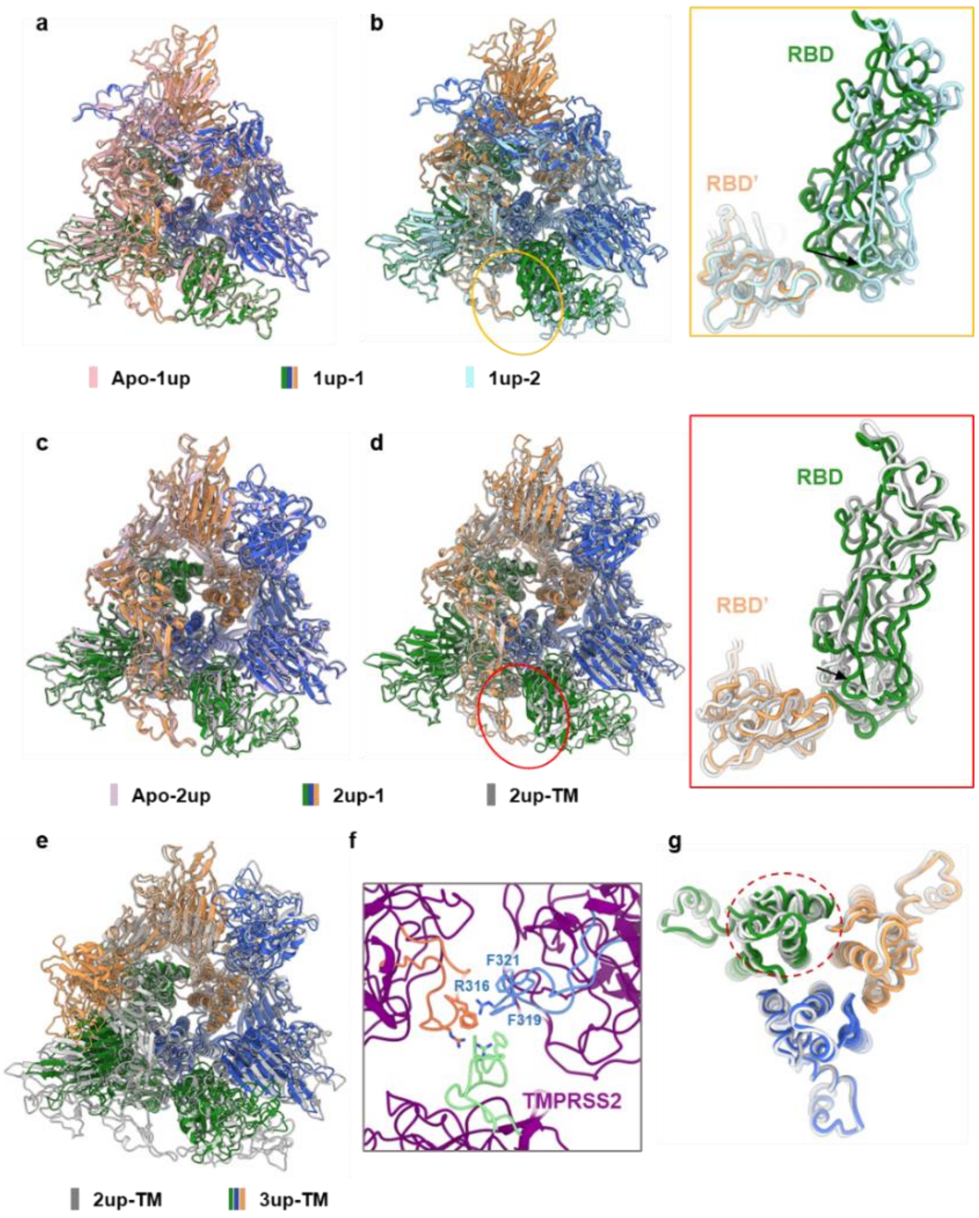

Fig. S11. Conformational changes induced by binding of HKU1-B S protein to receptor TMPRSS2.

a, Comparison of the S protein between 1up conformation and 1up-1 conformation. **b,** Comparison of the S protein between 1up-2 conformation and 1up-1 conformation. The RBDs interface changes between protomer 1 and adjacent protomer 3 are magnified in the orange box. Black arrows indicate the direction of movement. **c,** Comparison of the S protein between 2up conformation with 2up-1 conformation. **d,** Comparison of the S protein between 2up-1 conformation and 2up-TMR conformation. Black arrows indicate the direction of movement. The conformational changes of RBD interface between protomer 1 and adjacent protomer 3 (red ellipse) are magnified in the red box. **e,** Comparison of the S protein between 2up-TMR conformation and 3up-TMR conformation. **f,** Interface of TMPRSS2 trimer of 3up-TMR conformation. **g,** Conformational changes in the CH region of S2 subunit in 3up-TMR conformation.

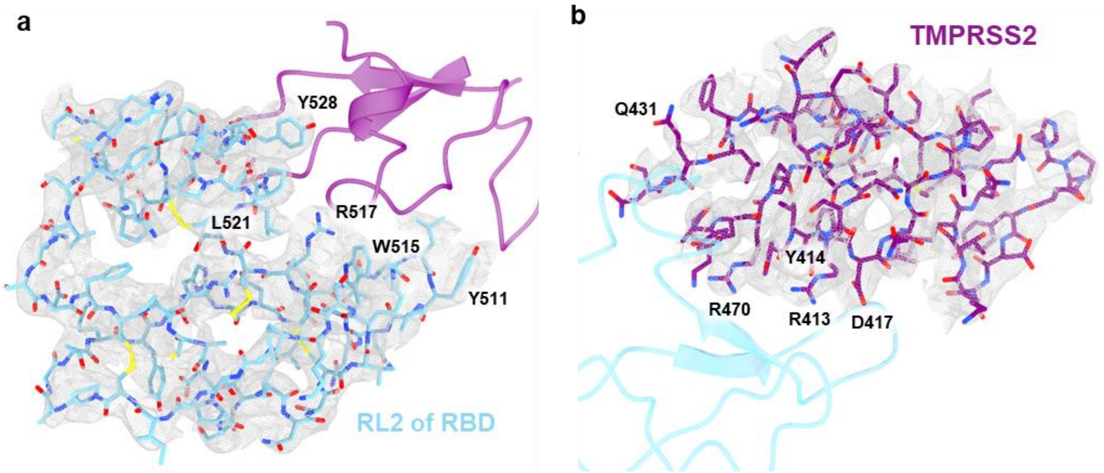

Fig. S12. Cryo-EM maps of the interface between HKU1-B RBD and TMPRSS2.

Cryo-EM maps of the HKU1-B RBD (**a**) and TMPRSS2 (**b**), which were displayed at threshold of 9 σ.

16.

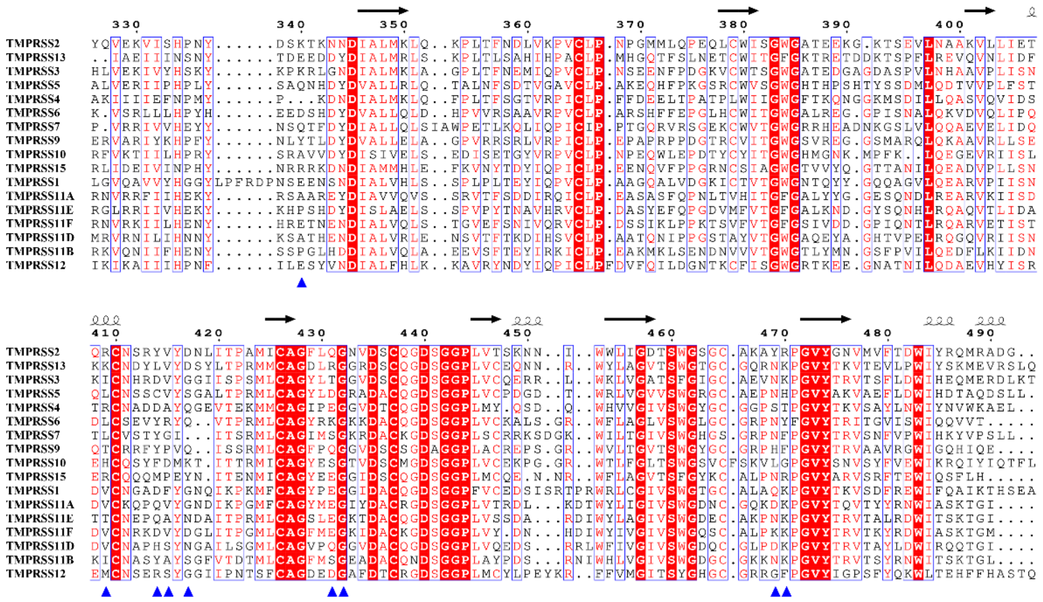

Fig. S13. Sequence alignment of TMPRSS family proteins

The blue triangle subscript indicates residues on TMPRSS2 that interact with the HKU1-B S protein.

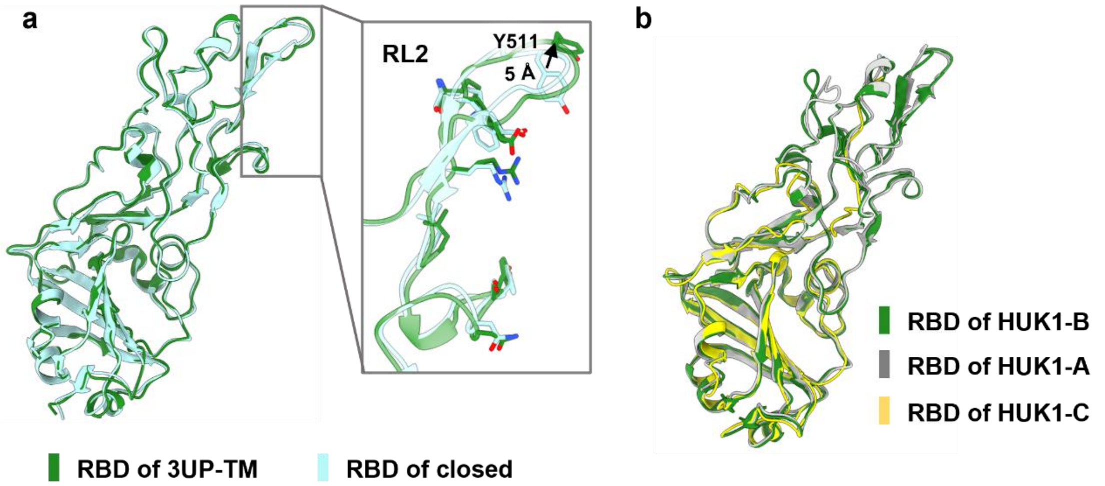

Fig. S14. Comparison of RBD structures of HKU1 S protein.

a, Conformational differences in HKU1-B RBD before and after binding to receptor TMPRSS2. **b,** Structural comparison of RBDs in different subtypes of HKU1 S protein.

Supplementary information, Table 1. Cryo-EM data collection and refinement statistics of HKU1-B S protein in the apo state.

**Data collection**			
EM equipment	Titan Krios (Thermo Fisher Scientific)		
Voltage (kV)	300		
Detector	Gatan K3 Summit		
Energy filter	Gatan GIF Quantum, 20 eV slit		
Pixel size (Å)	1.087		
Electron dose (e-/Å2)	50		
Defocus range (μm)	-1.2 ~ -2.2		
Number of collected micrographs	1,452		
Sample	Closed	1up	2up
PDB ID	8Y1F	8Y1G	8Y1H
EMDB ID	EMD-38834	EMD-38835	EMD-38836
**3D Reconstruction**			
Software	cryoSPARC	cryoSPARC	cryoSPARC
Number of used particles	104,714	148,802	134,669
Resolution (Å)	2.80	2.99	3.16
Symmetry		C1	
Map sharpening B factor ($Å^2$)		-90	
**Refinement**			
Software		Phenix	
Cell dimensions (Å)			
a=b=c (Å)		434.8	
α=β=γ (˚)		90	
Model composition			
Protein residues	3,624	3,624	3,624
Sugar	93	93	93
R.m.s deviations			
Bonds length (Å)	0.004	0.006	0.010
Bonds Angle (˚)	0.684	0.785	1.054
Ramachandran plot statistics (%)			
Preferred	95.33	93.84	91.96
Allowed	4.64	6.08	8.02
Outlier	0.03	0.08	0.03

Supplementary information, Table 2. Cryo-EM data collection and refinement statistics of the incubation of HKU1-B S protein with TMPRSS2.

**Data collection**						
EM equipment	Titan Krios (Thermo Fisher Scientific)					
Voltage (kV)	300					
Detector	Gatan K3 Summit					
Energy filter	Gatan GIF Quantum, 20 eV slit					
Pixel size (Å)	1.087					
Electron dose (e-/Å2)	50					
Defocus range (μm)	-1.2 ~ -2.2					
Number of collected micrographs	5,113					
Sample	Closed	1up-1	1up-2	2up-1	2up-TM	3up-TM
PDB ID	8Y19	8Y1A	8Y1B	8Y1C	8Y1D	8Y1E
EMDB ID	EMD-38828	EMD-38829	EMD-38830	EMD-38831	EMD-38832	EMD-38833
**3D Reconstruction**						
Software	cryoSPARC	cryoSPARC	cryoSPARC	cryoSPARC	cryoSPARC	cryoSPARC
Number of used particles	622,548	150,172	457,554	212,902	408,603	366,277
Resolution (Å)	2.30	2.80	2.80	2.80	2.70	2.70
Symmetry	C1					
Map sharpening B factor ($Å^2$)	-90					
**Refinement**						
Software	Phenix					
Cell dimensions<br>a=b=c (Å)<br>α=β=γ (˚)	 434.8<br>90					
Model composition						
Protein residues	3,624	3,624	3,624	3,624	4,317	4,659
Sugar	93	93	93	93	93	93
R.m.s deviations						
Bonds length (Å)	0.006	0.006	0.005	0.007	0.006	0.010
Bonds Angle (˚)	0.866	0.823	0.770	0.879	0.863	1.020
Ramachandran plot statistics (%)						
Preferred	95.16	93.15	94.72	92.65	93.15	91.29
Allowed	4.78	6.80	5.26	7.3	6.83	8.62
Outlier	0.06	0.06	0.00	0.06	0.03	0.08

Supplementary information, Video S1

Conformational change of the HKU1-B S protein from closed status to 1up and 2up status.

Supplementary information, Video S2

Conformational change of the (sub)domains of HKU1-B S protein from closed status to 1up status.

Supplementary information, Video S3

Conformational change of the HKU1-B S protein from 1up-2 status to 2up-TMR and 3up-TMR status.
